# ERGA-BGE genome of *Dailognatha quadricollis, *an East Mediterranean darkling beetle

**DOI:** 10.12688/openreseurope.20840.1

**Published:** 2025-08-06

**Authors:** Giannis Bolanakis, Danae Karakasi, Eleftherios Bitzilekis, Manos Stratakis, Apostolos Trichas, Petros Lymberakis, Nikos Poulakakis, Astrid Böhne, Rita Monteiro, Rosa Fernández, Nuria Escudero, Genoscope Sequencing Team, Manon Angel, Jean-Marc Barbance, Julie Batisse, Odette Beluche, Laurie Bertrand, Elodie Brun, Maria Dubois, Corinne Dumont, Barbara Estrada, Thomas Guerin, Zineb El Hajji, Sandrine Lebled, Patricia Lenoble, Claudine Louesse, Ghislaine Magdelenat, Eric Mahieu, Claire Milani, Sophie Oztas, Marine Paillard, Emilie Payen, Emanuelle Petit, Murielle Ronsin, Benoit Vacherie, Alice Moussy, Corinne Cruaud, Karine Labadie, Lola Demirdjian, Emilie Téodori, Patrick Wincker, Pedro H. Oliveira, Jean-Marc Aury, Chiara Bortoluzzi

**Affiliations:** 1 Department of Biology, School of Sciences and Engineering, Vassilika Vouton, University of Crete, Heraklion, GR-70013, Greece; 2 Natural History Museum of Crete, School of Sciences and Engineering, Knossos Avenue, University of Crete, Heraklion, GR-71409, Greece; 3 Foundation for Research and Technology – Hellas (FORTH), Institute of Molecular Biology and Biotechnology (IMBB),, Heraklion, GR-70013, Greece; 4 Leibniz Institute for the Analysis of Biodiversity Change, Adenauerallee 127, Museum Koenig Bonn, Bonn, 53113, Germany; 5 Metazoa Phylogenomics Lab, Passeig marítim de la Barceloneta 37-49, Institute for Evolutionary Biology (CSIC-UPF), Barcelona, 08003, Spain; 6 Genoscope, Institut François Jacob, CEA, CNRS, Univ Evry, Université Paris-Saclay, Evry, 91057, France; 7 Génomique Métabolique, Genoscope, Institut François Jacob, CEA, CNRS, Univ Evry, Université Paris-Saclay, Evry, 91057, France; 8 SIB Swiss Institute of Bioinformatics, Amphipôle, Quartier UNIL-Sorge, Lausanne, 1015, Switzerland

**Keywords:** Dailognatha quadricollis, genome assembly, European Reference Genome Atlas, Biodiversity Genomic Europe, Earth Biogenome Project, Tenebrionidae

## Abstract

*Dailognatha quadricollis* (Coleoptera: Tenebrionidae) is a darkling beetle native to the Balkans and Eastern Mediterranean, with a range extending from Croatia to Lebanon. It is a morphologically diverse species, comprising numerous subspecies, particularly concentrated in the Aegean region. The reference genome of *Dailognatha quadricollis* will enable phylogenetic, population and evolutionary research. The entirety of the genome sequence was assembled into 11 contiguous chromosomal pseudomolecules (sex chromosome included). This chromosome-level assembly encompasses 0.52 Gb, composed of 393 contigs and 322 scaffolds, with contig and scaffold N50 values of 4.3 Mb and 23.6 Mb, respectively.

## Introduction

*Dailognatha quadricollis* (Coleoptera: Tenebrionidae) is a darkling beetle found in the Balkans and the East Mediterranean area, with a distribution ranging from Croatia to Lebanon ( Iwan *et al.*, 2020). *Dailognatha quadricollis* is a highly diverse species characterised by numerous subspecies, most of which are in the Aegean region. The species is quite widespread and abundant and is mostly found in the Mediterranean shrublands, such as phrygana and maquis. *Dailognatha quadricollis* is classified by the Red Data Book of Greece ( NECCA, 2024) as Least Concern. The species feeds on seeds, roots and plant debris, and contributes to plant matter decomposition.

The reference genome of *Dailognatha quadricollis* will aid in understanding the species' phylogeny, taxonomic structure, and evolutionary adaptations, as well as those of its close relatives. The generation of this reference resource was coordinated by the European Reference Genome Atlas (ERGA) initiative’s Biodiversity Genomics Europe (BGE) project, supporting ERGA’s aims of promoting transnational cooperation to promote advances in the application of genomics technologies to protect and restore biodiversity ( Mazzoni *et al.*, 2023).

## Materials & Methods

ERGA's sequencing strategy includes Oxford Nanopore Technology (ONT) and/or Pacific Biosciences (PacBio) for long-read sequencing, along with Hi-C sequencing for chromosomal architecture, Illumina Paired-End (PE) for polishing (i.e. recommended for ONT-only assemblies), and RNA sequencing for transcriptome profiling, to facilitate genome assembly and annotation.

### Sample and sampling information

**Figure 1.**
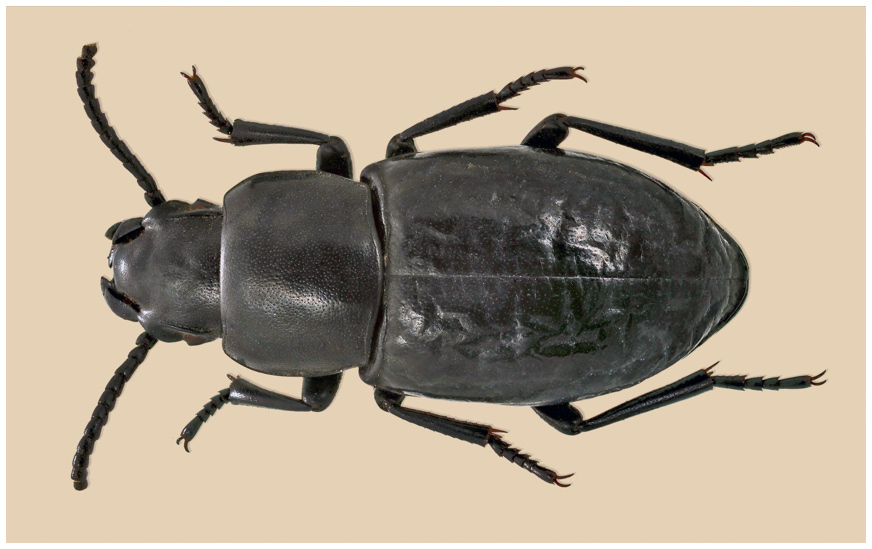
A male specimen of the species *Dailognatha quadricollis*. The individual in the picture is from the island of Crete and is a different specimen from the one sampled and sequenced for this study. Photo credit: Dr Apostolos Trichas.

On 04 June 2023, Giannis Bolanakis sampled 7 adult specimens (sex unknown) of *Dailognatha quadricollis*, which were identified by the expert Apostolos Trichas ( Figure 1). The specimens were sampled in Psiloritis, Lochria, Akolita, Heraklion, Crete. Sampling was performed under Presidential Decree 67/81 issued by the Greek government. Specimens were hand-picked, euthanized in liquid nitrogen and, until DNA extraction, were preserved at -80 °C.

### Vouchering information

Physical reference materials have been deposited in the Arthropods Collections of the Natural History Museum of Crete (NHMC) of the University of Crete https://www.nhmc.uoc.gr/en/departments/arthropods/ under proxy voucher ID NHMC.85.2.19030.

Frozen reference tissue material is available from a proxy voucher at the Genomics and Genetic Resources Division of the NHMC https://www.nhmc.uoc.gr/en/departments/genomics under proxy voucher IDs NHMC.85.2.19030.1 and NHMC.85.2.19030.2.

### Genetic information

The estimated genome size, based on ancestral taxa, is 0.26 Gb. This is a diploid genome with a haploid number of 10 chromosomes (2n=20) and unknown sex chromosomes. All information for this species was retrieved from Genomes on a Tree ( Challis *et al.*, 2023).

### DNA/RNA processing

DNA was extracted from a whole individual (100 mg) using a conventional CTAB extraction followed by a commercial purification using Qiagen Genomic tips (Qiagen). A detailed protocol is available on protocols.io ( https://www.protocols.io/view/hmw-dna-extraction-for-long-read-sequencing-using-bp2l694yzlqe/v1). DNA fragment size selection was performed using Short Read Eliminator (PacBio). Quantification was performed using a Qubit dsDNA HS Assay kit (Thermo Fisher Scientific) and integrity was assessed in a FemtoPulse system (Agilent). DNA was stored at 4 °C until usage.

RNA was extracted from a whole individual (70 mg) using a RNeasy Plus Universal Kit (Qiagen) following manufacturer instructions. Residual genomic DNA was removed with 6U of TURBO DNase (2 U/μl) (Thermo Fisher Scientific). Quantification was performed using a Qubit RNA HS Assay and integrity was assessed in a Bioanalyzer system (Agilent). RNA was stored at -80° C.

### Library preparation and sequencing

Long-read DNA libraries were prepared with SMRTbell prep kit 3.0 following manufacturers' instructions and sequenced on a Revio system (PacBio). Hi-C libraries were generated from 2 different individuals using the Arima High Coverage HiC kit (following the Animal Tissues low input protocol v01) and sequenced on a NovaSeq 6000 instrument (Illumina) with 2x150 bp read length. Poly(A) RNA-Seq libraries were constructed using the Illumina Stranded mRNA Prep, Ligation Prep kit (Illumina) and sequenced on an Illumina NovaSeq 6000 instrument (Illumina).

In total 42x HiFi and 177x HiC data were sequenced to generate the assembly.

### Genome assembly methods

The genome of *Dailognatha quadricollis* was assembled using the Genoscope GALOP pipeline ( https://workflowhub.eu/workflows/1200). Briefly, raw PacBio HiFi reads and Hi-C data were assembled using Hifiasm v0.19.5-r593 ( Cheng *et al.*, 2021). The phased assembly was scaffolded using YaHS v1.2 ( Zhou *et al.*, 2023) and the resulting scaffolds were curated through manual inspection using PretextView v0.2.5 to remove false joins and to incorporate sequences not automatically scaffolded into their respective locations within the chromosomal pseudomolecules. Chromosome-scale scaffolds confirmed by Hi-C data were named in order of size and, for each chromosome, the longest haplotype was retained as the main haplotype. Additionally, the X chromosome was identified by comparison with the reference genome of *Tenebrio molitor* (icTenMoli1.1). The mitochondrial genome was assembled using Oatk v1.0 ( Zhou *et al.*, 2024) and included in the released assembly. Summary analysis of the released assembly was performed using the ERGA-BGE Genome Report ASM Galaxy workflow ( https://doi.org/10.48546/workflowhub.workflow.1104.1).

## Results

### Genome assembly

**Figure 2.**
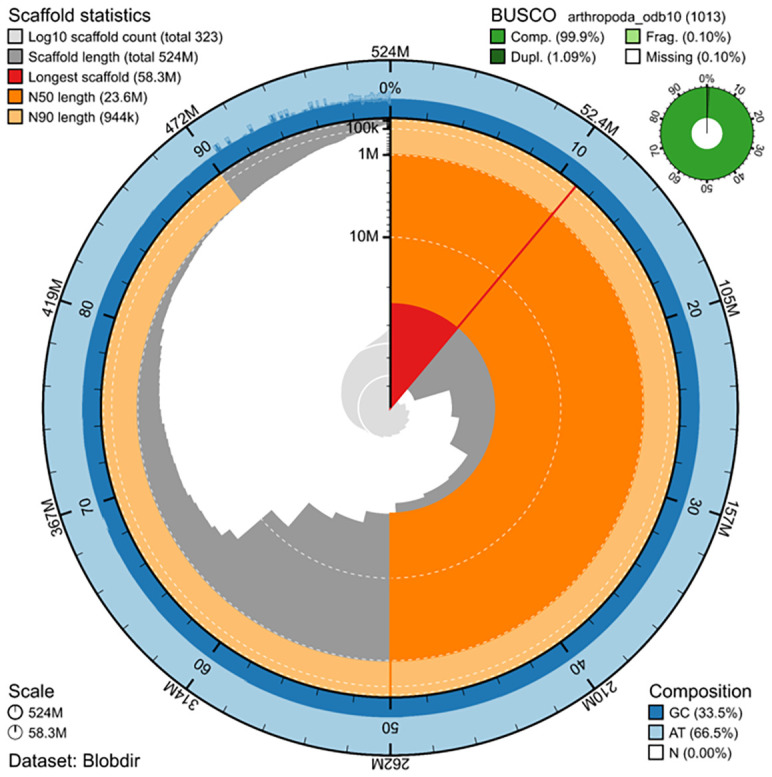
Snail plot summary of assembly statistics. The main plot is divided into 1,000 size-ordered bins around the circumference, with each bin representing 0.1% of the 524,088,546 bp assembly including the mitochondrial genome. The distribution of sequence lengths is shown in dark grey, with the plot radius scaled to the longest sequence present in the assembly (58.3 Mb, shown in red). Orange and pale-orange arcs show the scaffold N50 and N90 sequence lengths (23,571,702 and 943,584 bp), respectively. The pale grey spiral shows the cumulative sequence count on a log-scale, with white scale lines showing successive orders of magnitude. The blue and pale-blue area around the outside of the plot shows the distribution of GC, AT, and N percentages in the same bins as the inner plot. A summary of complete, fragmented, duplicated, and missing BUSCO genes found in the assembled genome from the Arthropoda database (odb10) is shown in the top right.

**Figure 3.**
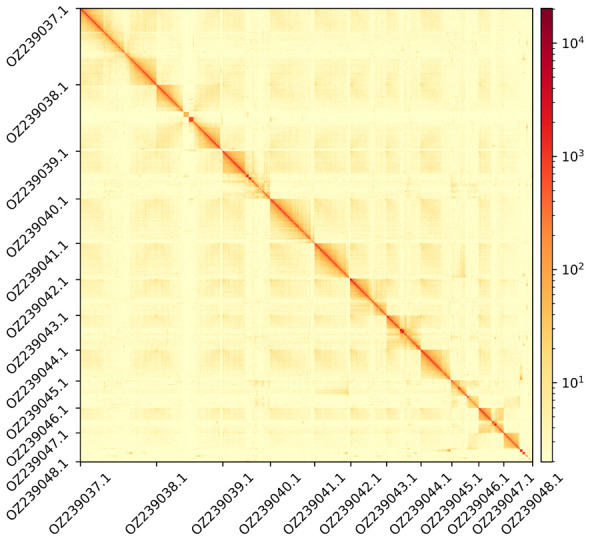
Hi-C contact map showing spatial interactions between regions of the genome. The diagonal corresponds to intra-chromosomal contacts, depicting chromosome boundaries. The frequency of contacts is shown on a logarithmic heatmap scale. Hi-C matrix bins were merged into a 100 kb bin size for plotting. The Hi-C map shows the autosomes, the X chromosome (GenBank: OZ239047.1), and the mitochondrial genome (OZ239048.1).

The genome assembly has a total length of 524,088,546 bp in 322 scaffolds including the mitogenome ( Figure 2 & Figure 3), with a GC content of 33.5%. The assembly has a contig N50 of 4,327,000 bp and L50 of 27 and a scaffold N50 of 23,571,702 bp and L50 of 8. The assembly has a total of 71 gaps, totalling 13.8 kb in cumulative size. The single-copy gene content analysis using the Arthropoda database with BUSCO ( Manni *et al.*, 2021) resulted in 99.9% completeness (98.8% single and 1.1% duplicated). 80.4% of reads k-mers were present in the assembly and the assembly has a base accuracy Quality Value (QV) of 65.7 as calculated by Merqury ( Rhie *et al.*, 2020).

## Data Availability

*D. quadricollis* and the related genomic study were assigned to Tree of Life ID (ToLID) ‘icDaiQuad1’ and all sample, sequence, and assembly information are available under the umbrella BioProject PRJEB77131. The sample information is available at the following BioSample accessions: SAMEA114349676, SAMEA114349677, and SAMEA114349689. The genome assembly is accessible from ENA under accession number GCA_965183915.1. The genome will be annotated using the generated RNA-Seq data and will be made available through the Ensembl website ( https://projects.ensembl.org/erga-bge/). Sequencing data produced as part of this project are available from ENA at the following accessions: ERX12722160, ERX12722161, ERX12725796, ERX12722095, ERX12733446, and ERX14096383. Documentation related to the genome assembly and curation can be found in the ERGA Assembly Report (EAR) document available at https://github.com/ERGA-consortium/EARs/tree/main/Assembly_Reports/Dailognatha_quadricollis/icDaiQuad1. Further details and data about the project are hosted on the ERGA portal at https://portal.erga-biodiversity.eu/data_portal/575809.

## Author information

Members of the Genoscope Sequencing Team are listed here: https://zenodo.org/records/14611490.
