# Supplementary material for: ERGA-BGE genome of Dailognatha quadricollis, an East Mediterranean darkling beetle
Source: Open Res Eur. 2026 May 29;5:225. Originally published 2025 Aug 6. [Version 2] doi: 10.12688/openreseurope.20840.2 (PMC13247569; doi:10.12688/openreseurope.20840.2)
Supplement: Peer review history

# Reviewer report: Reviewer response for version 2

**DOI:** 10.21956/openreseurope.26047.r75161

**Published:** 2026-06-08

**Authors:** Yingao Cai

**Affiliations:** 1 State Key Laboratory of Animal Biodiversity Conservation and Integrated Pest Management, Institute of Zoology Chinese Academy of Sciences, Chaoyang, Beijing, China

I would like to thank the authors for their detailed and thorough responses to my previous comments. The authors have addressed all of my concerns adequately, and the manuscript has been significantly improved. I have no further comments to make and am pleased to recommend the article for indexing.

Are sufficient details of methods and materials provided to allow replication by others?

Partly

Is the rationale for creating the dataset(s) clearly described?

Yes

Are the datasets clearly presented in a useable and accessible format?

Yes

Are the protocols appropriate and is the work technically sound?

Yes

Reviewer Expertise:

Genome assembly, transposable elements evolution, mutation process

I confirm that I have read this submission and believe that I have an appropriate level of expertise to confirm that it is of an acceptable scientific standard.

# Reviewer report: Reviewer response for version 1

**DOI:** 10.21956/openreseurope.22548.r62930

**Published:** 2025-11-13

**Authors:** Yingao Cai, Fengling Huang

**Affiliations:** 1 State Key Laboratory of Animal Biodiversity Conservation and Integrated Pest Management, Institute of Zoology Chinese Academy of Sciences, Chaoyang, Beijing, China

The article successfully sequencing and assembled the genome of the beetle *Dailognatha quadricollis*, achieving the high continuous and high accuracy (N50 of contigs: 4.3M and QV 65.7). The authors used both third-generation (TGS) and next-generation sequencing (NGS) technologies, along with chromosome-level sequencing methods, to generate the whole-genome sequncing data. They then applied a well-established genome assembly pipeline to construct contigs and scaffolds. While this research provides a valuable genomic resource for a non-model insect, its conclusions remain limited by several methodological and biological constraints.

 Major Concerns:

1. Traditional sex chromosome identification is based on the sequencing depth which the sequencing reads mapping to the contig or chromosomes difference between the male samples and female samples ^1, 2^. However, the specimens used in this study do not contain any information about the sex of the individuals. If the sequencing data had included male samples, the authors could have conducted additional analyses to identify and characterize sex chromosomes.
2. The mitochondrial genome and other cytoplasmic DNA like symbiotic bacteria genome can often be assembled as separate contigs using hifiasm. Did the authors find any contigs in the raw hifiasm output correspond to the results of Oatk.
3. The genome size estimated from ancestral taxa in GoaT is 0.26 Gb, while the expected haploid genome size reported on the ERGA assembly GitHub page is 415,868,280 bp. These values are inconsistent with each other. To determine the real genome size, author need to remove the assembly contaminations. Tools such as BlobTools2 can be used to identify and filter out sequences originating from other organisms.

Minor Concerns:

1. ONT and RNA sequencing data seem not applicated in the genome assembly, modified the Materials & Methods parts.

Are sufficient details of methods and materials provided to allow replication by others?

Partly

Is the rationale for creating the dataset(s) clearly described?

Yes

Are the datasets clearly presented in a useable and accessible format?

Yes

Are the protocols appropriate and is the work technically sound?

Yes

Reviewer Expertise:

Genome assembly, transposable elements evolution, mutation process

We confirm that we have read this submission and believe that we have an appropriate level of expertise to confirm that it is of an acceptable scientific standard, however we have significant reservations, as outlined above.

# Response

**Published:** 2026-05-18

**Authors:** Chiara Bortoluzzi

**Affiliations:** Swiss Institute of Bioinformatics, Lausanne, Vaud, Switzerland

We thank the reviewer for their comments. We have addressed them as follow:

1. As specified in the abstract, we were able to assemble only the X chromosome. We provided additional information about the sex mechanisms of the species in the Genetic information section. 
2. Yes, we were able to assemble the mitochondrial genome, which has been assigned GenBank accession ID OZ239048.1. 
3. We understand the misunderstanding. The genome size estimated from ancestral taxa in GoaT was mainly used to have an estimate of the amount of sequencing required for genome assembly. It is not meant to represent the actual genome size, which is indeed better estimated with other methods. 
4. We removed the ONT and RNA reference from the main text.

# Reviewer report: Reviewer response for version 1

**DOI:** 10.21956/openreseurope.22548.r62936

**Published:** 2025-11-06

**Authors:** Arun Arumugaperumal

**Affiliations:** 1 Rajalakshmi Engineering College, Chennai, Tamil Nadu, India

The data note describes the whole genome sequence of a beetle, *Dailognatha quadricollis*. There is not much literature present regarding the beetle. This is the first high-quality genome sequence of the beetle to be reported. The genome sequence reported herewith will be a valuable resource to study its phylogeny and explain the nature of the beetle, on analysis. The authors have used long-read sequencing and Hi-C data to arrive at a chromosome-level assembly.

 The assembly reported here is of size 0.52 Gb spread among 11 chromosomes. However, the authors are not sure about the sex of the sample. They had identified an X chromosome on comparison with the genome of a related beetle. The contig N50 of 4.3 Mb and scaffold N50 of 23.6 Mb indicate the good quality of the genome in terms of truncated sequences. It will be further evident once the annotation has been released in Ensembl. BUSCO analysis also showed that the genome has 99.9% completeness with respect to odb10 database.

 The mitochondrial genome has also been separated from the chromosomal sequences. However, not much information about it has been reported in the manuscript. The links provided for the datasets and the accession IDs are active. The data note can be indexed.

Are sufficient details of methods and materials provided to allow replication by others?

Yes

Is the rationale for creating the dataset(s) clearly described?

Yes

Are the datasets clearly presented in a useable and accessible format?

Yes

Are the protocols appropriate and is the work technically sound?

Yes

Reviewer Expertise:

Bioinformatics; Genomics

I confirm that I have read this submission and believe that I have an appropriate level of expertise to confirm that it is of an acceptable scientific standard.

# Response

**Published:** 2025-11-06

**Authors:** Chiara Bortoluzzi

**Affiliations:** Swiss Institute of Bioinformatics, Lausanne, Vaud, Switzerland

We thank the reviewer for their positive feedback. We would like to higlight that the gene annotation for *Dailognatha quadricollis *is now available in the Ensembl website: https://projects.ensembl.org/erga-bge/.
